# BAP31 Drives Cartilage Calcification through Disruption of Autophagosome–Lysosome Fusion in Osteoarthritis

**DOI:** 10.1002/advs.202520140

**Published:** 2026-08-29

**Authors:** Zhi‐hua Xu, Ruo‐xin Wang, Feng He, Qian Liu, Fu‐yin Li, Min Hui, Yu‐qian Shi, Yu Jiang, Qing‐hua Li, Jun‐wen Jing, Shi‐bin Yu, Li‐tian Ma, Qian‐nan Niu, Mian Zhang

**Affiliations:** ^1^ State Key Laboratory of Oral & Maxillofacial Reconstruction and Regeneration, National Clinical Research Center for Oral Diseases, Shaanxi International Joint Research Center For Oral Diseases, Department of Oral Anatomy and Physiology, School of Stomatology The Fourth Military Medical University Xi'an China; ^2^ State Key Laboratory of Oral & Maxillofacial Reconstruction and Regeneration, National Clinical Research Center for Oral Diseases, Shaanxi Clinical Research Center for Oral Diseases, Department of Orthodontics, School of Stomatology The Fourth Military Medical University Xi'an China; ^3^ State Key Laboratory of Oral & Maxillofacial Reconstruction and Regeneration, National Clinical Research Center for Oral Diseases, Shaanxi Key Laboratory of Stomatology, Department of Oral and Maxillofacial Surgery, School of Stomatology The Fourth Military Medical University Xi'an Shaanxi China; ^4^ Department of Stomatology, Air Force Medical Center Air Force Medical University, PLA Beijing China; ^5^ School of Medicine Northwest University Xi'an Shaanxi China; ^6^ Department of Pharmacology Shaanxi University of Chinese Medicine Xianyang Shaanxi China; ^7^ Department of Thoracic Surgery, Tangdu Hospital Air Force Medical University Xi'an Shaanxi China; ^8^ Department of Traditional Chinese Medicine, Tangdu Hospital Air Force Medical University Xi'an Shaanxi China

**Keywords:** autophagy, BAP31, exosome, osteoarthritis, pathological calcification

## Abstract

Pathological cartilage calcification represents a pivotal pathological alteration in osteoarthritis (OA), yet its underlying mechanisms remain poorly understood. Exosomes are involved in facilitating calcification, and their biogenesis may be linked to autophagy disruption. However, whether autophagy disruption regulates exosome‐mediated calcification in OA remains to be elucidated. Here, B‐cell receptor‐associated protein 31 (BAP31) was identified as a key mechanosensitive regulator bridging this gap. Mechanical stimulation activated BAP31 in chondrocytes. Protein docking, coimmunoprecipitation assays, and competitive pull‐down assays demonstrated that activated BAP31 competes with autophagy‐related 14 (ATG14) for binding to syntaxin 17 (STX17), thereby disrupting the STX17–ATG14 complex, which is essential for autophagosome‒lysosome fusion. This disruption led to the accumulation of autophagosomes, which in turn drove the release of exosomes that facilitated pathological cartilage calcification. Critically, intra‐articular delivery of adeno‐associated virus‐mediated BAP31 shRNA restored autophagosome‒lysosome fusion, suppressed exosome release, and attenuated cartilage calcification and degeneration in a rat OA model. Collectively, these findings identify BAP31 as a mechanical stress‐responsive switch that couples impaired autophagy to exosome‐mediated calcification, highlighting its potential as a therapeutic target for OA.

## Graphical abstract

Mechanical stress activates BAP31 in chondrocytes. BAP31 competes with ATG14 for binding to STX17, disrupting the STX17–ATG14 complex required for autophagosome–lysosome fusion. The resultant autophagic flux blockade drives the generation of autophagy‐derived exosomes, which mediate pathological cartilage calcification in OA. Chondrocyte‐targeted BAP31 silencing effectively restores autophagic homeostasis and attenuates cartilage calcification, representing a promising disease‐modifying strategy for OA treatment.

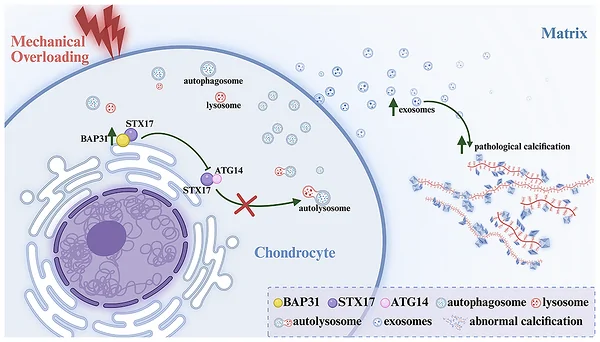

## 1 Introduction

Osteoarthritis (OA) is a multifactorial and progressive degenerative joint disease that affects approximately 7.6% of the global population, equating to nearly 595 million individuals worldwide [1]. It accounts for 2.3% of all‐cause years lived with disability, impacting approximately 21.3 million people worldwide [2]. Despite its significant health burden, no disease‐modifying therapies for OA are currently available. Mechanical overloading is a well‐established driver of OA pathogenesis [3], and pathological cartilage calcification represents a critical pathological change during this process [4, 5]. Cartilage calcification is detected in nearly all osteoarthritic joints and shows a strong correlation with disease severity [6]. Accumulating evidence demonstrates that calcification is not merely a passive consequence of OA degeneration but an active catalyst of disease progression, by altering cartilage biomechanical properties, inducing local inflammatory responses, and accelerating extracellular matrix degradation [4, 7, 8]. However, the mechanisms underlying mechanical stress‐induced pathological calcification in OA cartilage remain poorly understood.

Growing evidence indicates that cells in diverse pathophysiological contexts secrete extracellular vesicles (EVs) carrying cell‐state‐specific molecular cargoes, which participate in diverse physiological and pathological processes [9, 10, 11, 12, 13]. Notably, exosomes, a subclass of EVs measuring 30–150 nm in diameter and characterized by a phospholipid bilayer membrane [14], have been implicated in pathological calcification. Exosome cargo, which includes calcification precursors, facilitates ectopic calcification in various diseases, such as atherosclerosis and chronic kidney disease [9, 15, 16]. Our previous studies revealed an abundance of exosomes within calcified regions of OA cartilage [17]. Furthermore, pharmacological inhibition of exosome secretion has been shown to effectively attenuate pathological cartilage calcification in OA [17, 18, 19]. Moreover, evidence suggests that the accumulation of LC3^+^ exosomes derived from autophagosomes mediates pathological calcification in OA [20]. We previously demonstrated that disrupted autophagic flux was caused by impaired autophagosome‒lysosome fusion in osteoarthritic chondrocytes [21], leading us to hypothesize that LC3^+^ exosomes accumulate as a result of impaired autophagic flux. However, the precise mechanisms linking autophagic dysfunction to increased LC3^+^ exosome release in chondrocytes remain to be elucidated.

Syntaxin 17 (STX17) plays an essential role in autophagosome‒lysosome fusion by recruiting autophagy‐related 14 (ATG14) to form a functional complex that orchestrates membrane fusion [22, 23]. Disruption of the STX17–ATG14 interaction directly impairs fusion efficiency. Notably, B‐cell receptor‐associated protein 31 (BAP31), an endoplasmic reticulum scaffold protein and a newly identified binding partner of STX17, has been shown to promote autophagosome accumulation through competitive inhibition of STX17–ATG14 assembly [24, 25, 26, 27]. Although BAP31 is known to modulate autophagy, its role in the mechanical stress response, exosome‐mediated calcification, and OA progression has not been investigated.

In this study, we used a unilateral anterior crossbite (UAC) model in vivo and a fluid flow shear stress (FFSS) system in vitro to investigate the role of BAP31 in OA cartilage and its contribution to pathological cartilage calcification. Our findings establish BAP31 as a mechanosensitive switch that links impaired autophagy to exosome‐mediated calcification, highlighting BAP31 as a potential therapeutic target in osteoarthritis.

## 2 Results

### 2.1 Mechanical Stress Induces Exosome‐Mediated Pathological Calcification in OA Cartilage

**FIGURE 1:**
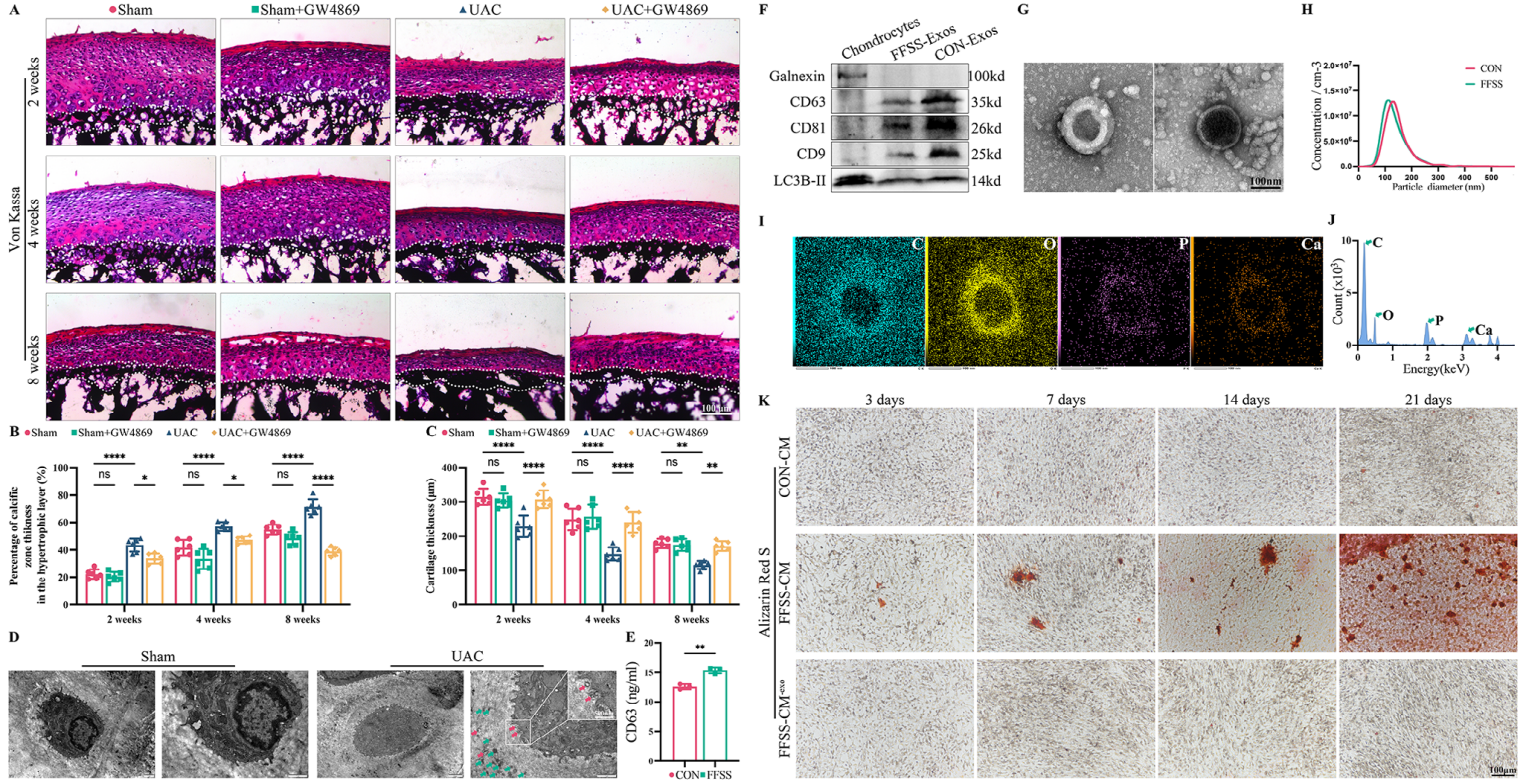
Mechanical stress induces exosome‐mediated pathological calcification in OA cartilage. (A) Representative Von Kossa staining images of condylar cartilage in the Sham, UAC, and UAC+GW4869 groups. (B) Quantification of the ratio of pathologically calcified zone to the entire hypertrophic layer in cartilage across groups (n = 6 animals per group). (C) Quantification of condylar cartilage thickness in different groups (n = 6). (D) Representative TEM images of pathological calcification in UAC‐treated rat condylar cartilage. The green arrows indicate sites of abnormal calcification, and the red arrows indicate exosomes. (E) Quantification of CD63 secretion in different groups (n = 3 independent experiments). (F) Western blot validation of exosome‐specific markers in CON‐exos and FFSS‐exos. (G) Representative TEM images showing the morphology of CON‐exos and FFSS‐exos. (H) Nanoparticle tracking analysis (NTA) showing the size distribution of CON‐exos and FFSS‐exos. (I) Elemental mapping of the exosomes derived from the FFSS group. J. Elemental analysis of (I). K. Time course analysis of matrix calcification induced by control cell‐derived conditioned medium (CON‐CM), FFSS‐treated cell‐derived CM (FFSS‐CM), or FFSS‐CM following exosome depletion (FFSS‐CM^−exos^) in ATDC5 cells, as visualized by Alizarin Red S staining. One‐way ANOVA and unpaired Student's t test were used. ns, not significant, ^*^ *p* < 0.05, ^**^ *p* < 0.01, and ^****^ *p* < 0.0001.

Given the established role of exosomes in pathological calcification in OA [18], we quantified calcification dynamics and verified the role of exosomes in a unilateral anterior crossbite (UAC) model. Von Kossa staining demonstrated that, compared with the Sham group, the UAC group had a greater percentage of hypertrophic chondrocytes undergoing pathological calcification at 2, 4, and 8 weeks (UAC: 43.5 ± 4.7%, 56.9 ± 3.4%, and 71.5 ± 5.7%; Sham: 22.3 ± 3.4%, 41.6 ± 5.6%, and 54.9 ± 3.6%, respectively; all *p* < 0.0001). Hematoxylin and Eosin staining (H&E staining) further showed that the UAC group exhibited significantly thinner condylar cartilage at 2, 4, and 8 weeks compared with the Sham group (UAC: 228.5 ± 31.4 µm, 147.9 ± 19.5 µm, and 115.6 ± 11.4 µm; Sham: 315.1 ± 23.8 µm, 249.0 ± 31.6 µm, and 178.5 ± 13.6 µm, respectively; at 2 and 4 weeks, *p* < 0.0001; at 8 weeks, *p* < 0.01). Importantly, intra‐articular injection of GW4869 markedly attenuated both the excessive cartilage calcification and cartilage thinning induced by UAC. However, administration of GW4869 alone to Sham‐operated rats (Sham+GW4869 group) did not significantly alter cartilage thickness or calcification percentage compared with the Sham group (all p > 0.05), confirming that GW4869 itself does not adversely affect normal cartilage homeostasis (Figure 1A–C; Figure S1). Transmission electron microscopy (TEM) of UAC cartilage showed clusters of 50–150 nm vesicles with typical exosomal morphology surrounding the chondrocytes. Furthermore, electron‐dense calcification foci were observed close to these vesicles, suggesting a possible spatial association between them (Figure 1D).

Western blotting (WB) revealed that fluid flow shear stress (FFSS) stimulation significantly upregulated the expression of CD63 (an exosome marker) in chondrocytes (Figure S2). Consistent with these findings, the level of CD63 protein in the supernatant of FFSS‐stimulated chondrocytes was significantly greater than that in the control chondrocytes (Figure 1E). The characteristic and function of exosomes derived from the control group (CON‐exos) and the FFSS group (FFSS‐exos) were further examined. Protein expression of exosome‐ specific markers (CD81, CD63, and CD9) was verified in both groups (Figure 1F). Compared with CON‐exos, FFSS‐exos were smaller in size and exhibited a more compact internal structure (Figure 1G,H). Elemental mapping analysis further revealed that FFSS‐exos contained detectable amounts of calcium and phosphorus, which were absent in CON‐exos (Figure 1I,J; Figure S3). To functionally assess the role of these exosomes in the calcification of chondrocytes, ATDC5 cells were incubated with conditioned medium (CM) from control chondrocytes (CON‐CM), FFSS‐stimulated chondrocytes (FFSS‐CM), or FFSS‐CM following exosome depletion (FFSS‐CM^−exos^). Alizarin red staining revealed that FFSS‐CM significantly promoted calcification in a time‐dependent manner. In contrast, both CON‐CM and FFSS‐CM^−exos^ had minimal effects (Figure 1K). Collectively, these in vivo and in vitro findings establish a direct link between mechanically induced exosome secretion from chondrocytes and the progression of pathological calcification.

### 2.2 Autophagy Disruption Triggers Exosome Biogenesis

**FIGURE 2:**
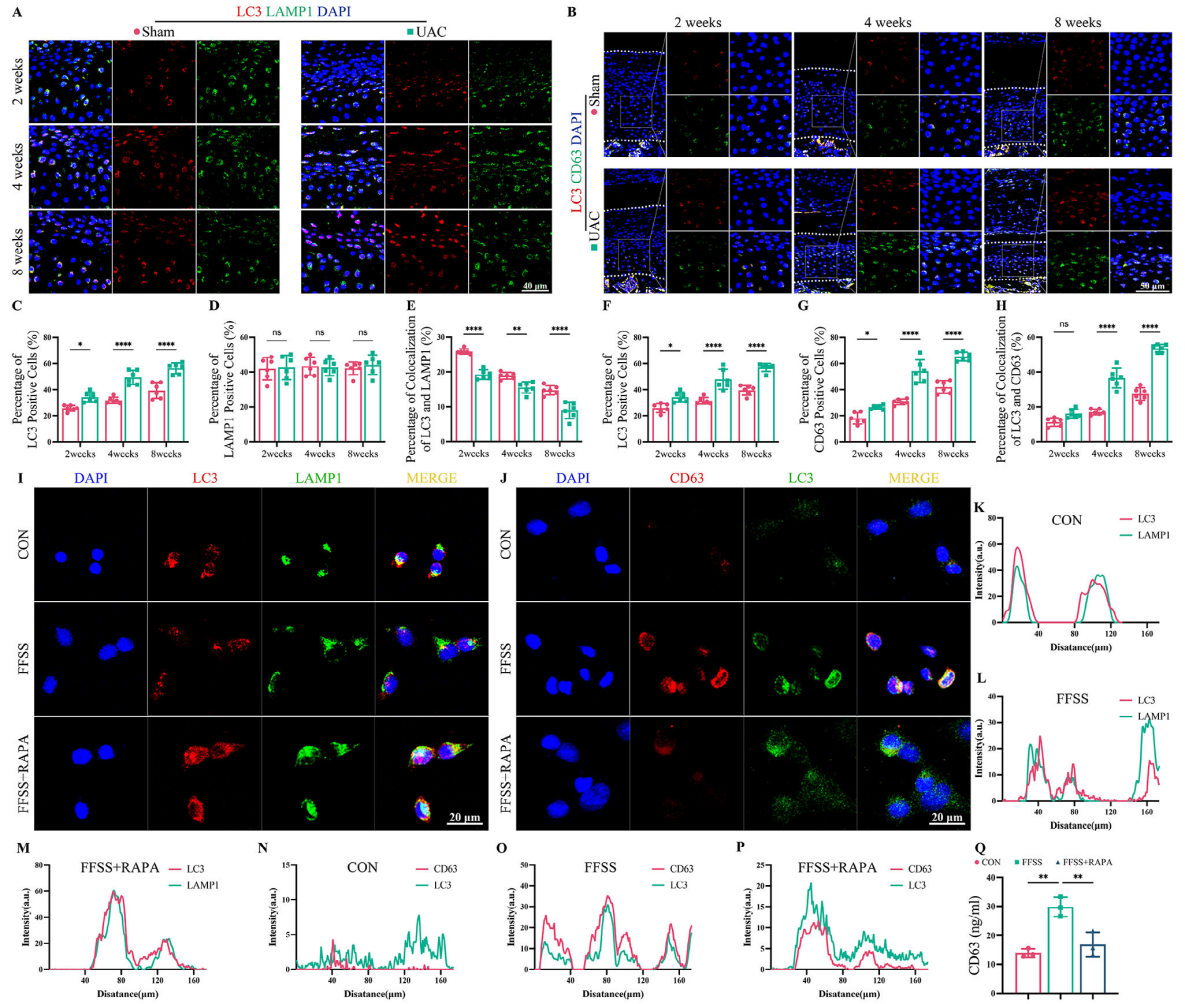
Disruption of autophagy triggers exosome biogenesis. (A) Representative IF images of LC3 and LAMP1 in condylar cartilage from different groups. (B) Representative IF images of LC3 and CD63 in condylar cartilage across groups. (C–E) Quantification of LC3^+^ cells, LAMP1^+^ cells, and LC3–LAMP1 colocalization percentages across groups (n = 6 animals per group). (F–H) Quantification of LC3^+^ cells, CD63^+^ cells, and LC3–CD63 colocalization percentages across groups (n = 6). (I) Representative IF images of LC3 and LAMP1 in ATDC5 cells across groups. (J) Representative IF images of LC3 and CD63 expression in ATDC5 cells across groups. (K–M) Quantification of LC3 and LAMP1 staining in cultured chondrocytes under (K) CON, (L) FFSS, and (M) FFSS+RAPA conditions. (N–P) Quantification of LC3 and CD63 staining in cultured chondrocytes under (N) CON, (O) FFSS, and (P) FFSS+RAPA conditions. (Q) Quantification of CD63 secretion in different groups (n = 3 independent experiments). One‐way ANOVA and unpaired Student's t test were used. ns, not significant, ^*^ *p* < 0.05, ^**^ *p* < 0.01, and ^****^ *p* < 0.0001.

To determine whether exosome‐mediated calcification is associated with autophagy disruption, we first performed dual immunofluorescence (IF) staining for LC3 (an autophagosome marker) and LAMP1 (a lysosome marker). Compared with the Sham group, the UAC group exhibited reduced colocalization of LC3 and LAMP1 (39.7% reduction at 8 weeks; *p* < 0.0001; Figure 2A,C–E), suggesting impaired autophagosome–lysosome association under pathological conditions. Given that accumulated autophagosomes may contribute to exosome biogenesis, we next analyzed the colocalization of LC3 and CD63 [20]. Dual IF staining revealed that UAC stimulation upregulated the expression of both proteins and markedly increased their colocalization (Figure 2B,F–H). These data suggest that autophagy impairment is accompanied by increased association between autophagosomes and exosome‐related compartments.

Consistent with the in vivo observations, similar alterations were observed in the in vitro FFSS model. Pearson correlation analysis revealed that LC3–LAMP1 colocalization was reduced following FFSS stimulation, with the correlation coefficient decreasing from r = 0.76 in control chondrocytes to r = 0.41 after FFSS treatment, indicating impaired autophagosome‐lysosome colocalization under mechanical stress. Importantly, restoration of autophagic activity by rapamycin (RAPA) treatment significantly recovered LC3–LAMP1 colocalization, with the Pearson correlation coefficient increasing to r = 0.70 in the FFSS+RAPA group, suggesting that activation of autophagy partially restores autophagosome–lysosome interaction (Figure 2I,K–M). In contrast, LC3–CD63 colocalization was low under control conditions (r = 0.22) but significantly increased following FFSS stimulation (r = 0.77), suggesting enhanced interaction between accumulated autophagosomes and exosome‐related compartments. However, LC3–CD63 colocalization was reduced following RAPA treatment (r = 0.60), suggesting that restoration of autophagic flux decreases the redistribution of autophagic components toward exosome‐related compartments (Figure 2J,N–P). To further determine whether autophagy disruption affects extracellular vesicle release, CD63 levels in culture supernatants were quantified by ELISA. FFSS stimulation significantly increased extracellular CD63 levels compared with control conditions, indicating enhanced release of CD63‐positive extracellular vesicles under mechanical stress. Conversely, RAPA treatment significantly reduced FFSS‐induced CD63 elevation, suggesting that restoration of autophagic activity suppresses excessive extracellular vesicle release associated with autophagy impairment (Figure 2Q). Furthermore, Alizarin Red S staining demonstrated that FFSS stimulation markedly promoted chondrocyte mineralization, as evidenced by increased calcium deposition, whereas RAPA treatment substantially alleviated this FFSS‐triggered calcification phenotype, providing direct evidence that pharmacological restoration of autophagy counteracts the pathological mineralization process (Figure S4).

To exclude the possibility that FFSS‐induced accumulation of LC3‐II and p62 resulted from impaired lysosomal degradation, cells were treated with bafilomycin A1 (BafA1) to fully inhibit lysosomal acidification. Compared with control cells, BafA1 treatment markedly increased LC3‐II and p62 accumulation, confirming effective blockade of autophagic degradation. FFSS stimulation also increased LC3‐II and p62 levels, suggesting that FFSS induces autophagosome accumulation and autophagic flux impairment. Moreover, combined FFSS and BafA1 treatment led to further accumulation of p62 and LC3‐II compared with FFSS stimulation alone, indicating that lysosomal degradative capacity was not fully saturated by mechanical stress and remained responsive to acidification inhibition. Notably, none of the above treatments altered LAMP1 expression in chondrocytes (Figure S5). These data indicate that FFSS‐induced autophagic flux blockade arises from disrupted autophagosome‐lysosome fusion, rather than altered lysosomal abundance or general lysosomal degradative dysfunction.

Next, we examined the characteristics of exosomes derived from control and FFSS‐stimulated chondrocytes. Western blot analysis confirmed the LC3B‐II/CD63 ratio was significantly higher in FFSS‐exos than in CON‐exos (Figure 1F; Figure S6), further supporting the notion that these exosomes originate, at least in part, from autophagosomal sources.

Taken together, these findings suggest that mechanical stress‐induced autophagy impairment is associated with reduced autophagosome–lysosome fusion and increased spatial association between autophagosomes and exosome‐related compartments, which may contribute to enhanced exosome biogenesis in OA.

### 2.3 The Autophagy‐Related STX17–ATG14 Interaction is Impaired by Mechanical Overloading

**FIGURE 3:**
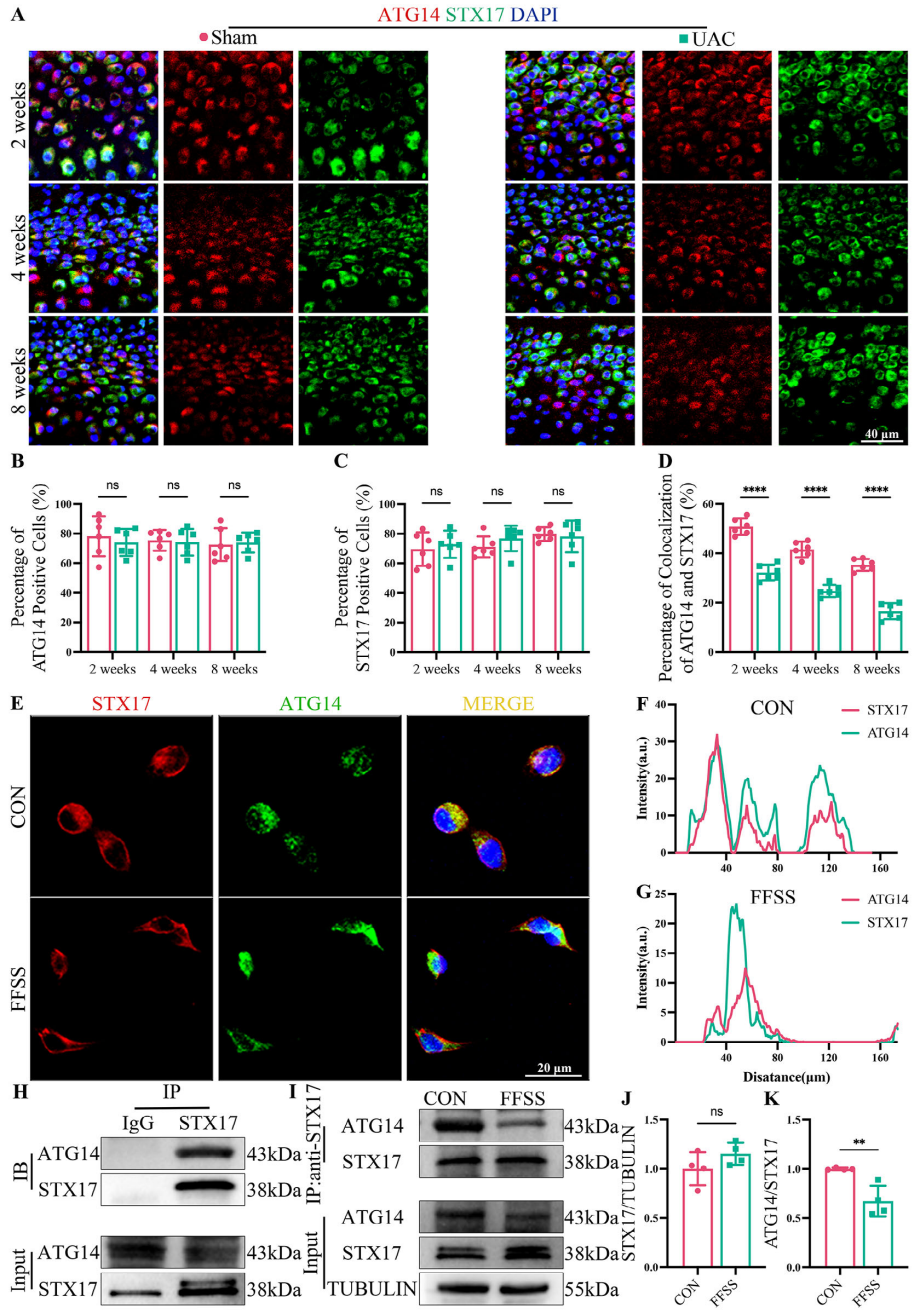
Autophagy‐related interactions between STX17 and ATG14 are impaired by mechanical overloading. (A) Representative IF images of ATG14 and STX17 in condylar cartilage from different groups. (B–D) Quantification of ATG14^+^ cells, STX17^+^ cells, and ATG14–STX17 colocalization percentages across groups (n = 6 animals per group). E. Representative IF images of ATG14 and STX17 in ATDC5 cells with or without FFSS stimulation. (F,G) Quantification of ATG14 and STX17 staining in cultured chondrocytes under (F) CON and (G) FFSS conditions. (H–K) Co‐IP was performed using anti‐STX17 and anti‐ATG14 antibodies to determine the interaction between STX17 and ATG14 in chondrocytes. (I) Quantification of STX17 enrichment levels in different groups by IP (n = 4 biological replicates). (J) Quantification of ATG14–STX17 interaction levels across different groups by IP (n = 4). One‐way ANOVA and unpaired Student's t test were used. ns not significant, ^**^ *p* < 0.01, and ^***^ *p* < 0.001.

Given that STX17 is essential for autophagosome‒lysosome fusion through the recruitment of ATG14 to form functional complexes that orchestrate the membrane fusion process [22, 23], we next investigated whether mechanical overloading impaired these functional interactions. Quantitative analysis revealed that UAC stimulation did not significantly affect the expression levels of ATG14 or STX17 (p > 0.05) but markedly decreased their colocalization by 36.5%, 40.4%, and 53.1% at 2, 4, and 8 weeks, respectively (*p* < 0.001; Figure 3A–D). Consistent with the in vivo findings, similar changes were observed in vitro. Pearson correlation analysis revealed a high degree of colocalization between ATG14 and STX17 in control chondrocytes (r = 0.78), which was markedly reduced following FFSS stimulation (r = 0.42), indicating disrupted spatial association between these proteins (Figure 3E–G). Notably, FFSS did not significantly affect the expression levels of ATG14 or STX17 (*p* > 0.05; Figure S7). Co‐immunoprecipitation (co‐IP) assays further showed that although STX17 expression remained unchanged, FFSS stimulation reduced the interaction between ATG14 and STX17, with binding efficiency decreased to 67.2% of that in the control group (Figure 3H–K).

### 2.4 Upregulation of BAP31 Expression by Mechanical Stress Blocks the Formation of the STX17–ATG14 Complex

To identify potential regulators that may influence STX17–ATG14 interaction and contribute to autophagy dysfunction in OA, we screened the Biological General Repository for Interaction Datasets (BioGRID) for STX17 interaction partners. The top five candidates included ATG14, C5AR2, PTPN2, SNAP29, and BCAP31 (BAP31) (Figure S8). Among these proteins, SNAP29 is a core component of the SNARE complex that facilitates autophagosome–lysosome fusion, and its recruitment is enhanced by ATG14 [28]. Given the limited evidence linking C5AR2 or PTPN2 to autophagy regulation, we focused on BAP31 for further investigation.

**FIGURE 4:**
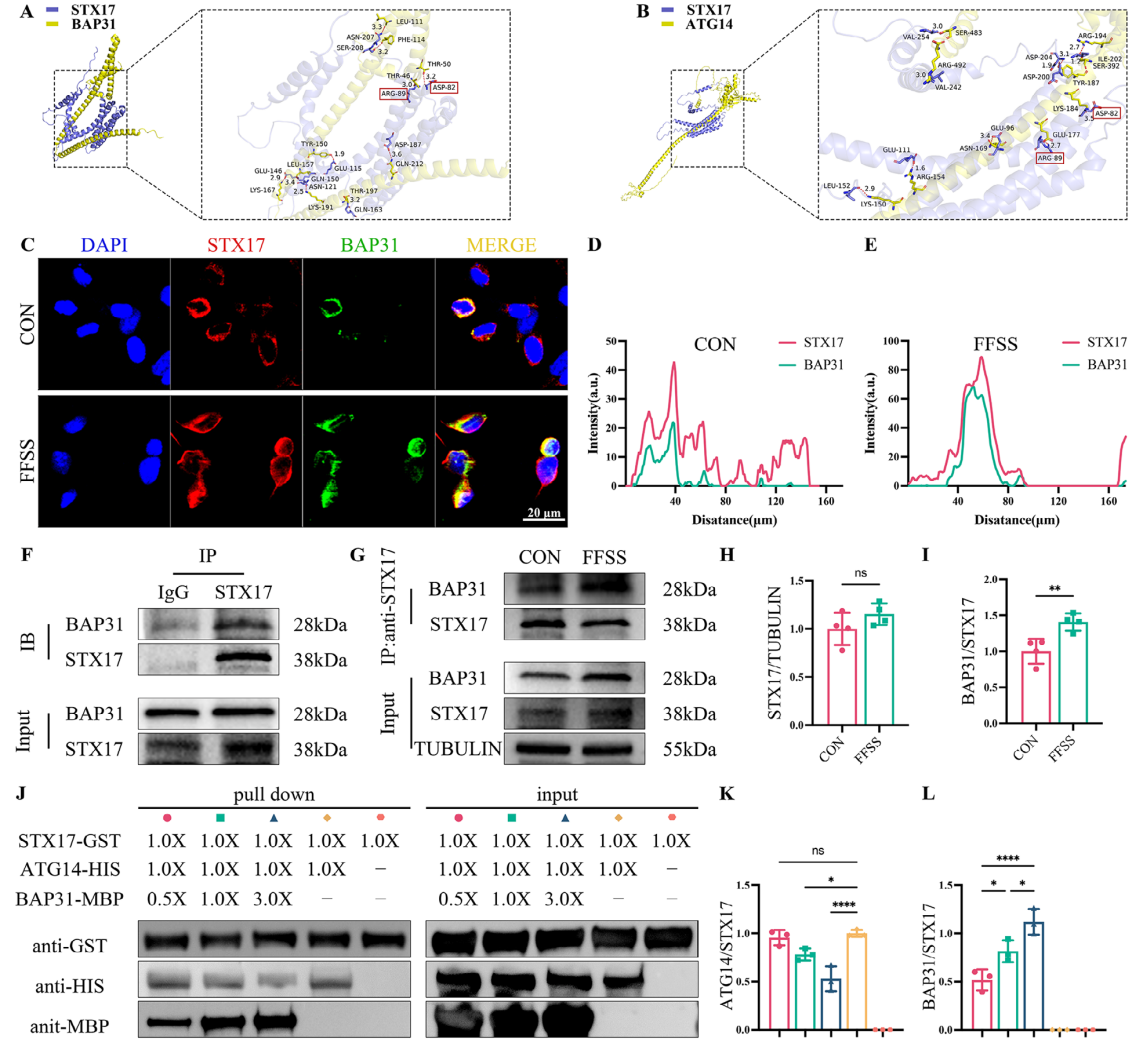
Upregulation of BAP31 expression by mechanical stress blocks STX17–ATG14 complex formation. (A) Computational docking model between BAP31 and STX17. (B) Computational docking model between ATG14 and STX17. (C) Representative IF images of BAP31 and STX17 in ATDC5 cells with or without FFSS stimulation. (D,E) Quantification of BAP31 and STX17 staining in cultured chondrocytes under (D) CON and (E) FFSS conditions. (F,G) Co‐IP was performed using anti‐STX17 and anti‐BAP31 antibodies to determine the interaction between STX17 and BAP31 in chondrocytes. (H) Quantification of STX17 enrichment levels in different groups by IP (n = 4 biological replicates). (I) Quantification of BAP31–STX17 interaction levels across different groups by IP (n = 4). (J) Pull‐down assays performed with constant STX17 and ATG14 levels and increasing BAP31 concentrations. (K) Quantification of ATG14–STX17 interaction levels in different groups by pull‐down assay (n = 3). (L) Quantification of BAP31–STX17 interaction levels in different groups by pull‐down assay (n = 3). Unpaired Student's t test and One‐way ANOVA. ns not significant, ^*^ *p* < 0.05, ^**^ *p* < 0.01, ^****^ *p* < 0.0001.

Molecular docking analysis revealed that both BAP31 and ATG14 share overlapping binding interfaces on STX17, with comparable binding energies (ΔG_bind: −24.3 kcal/mol and −24.8 kcal/mol, respectively) and approximately 60% overlap in critical interaction residues. Notably, aspartate‐82 (D82) and arginine‐89 (R89) of STX17 exhibited complete structural overlap at the predicted binding interface (Figure 4A,B). These findings suggest that BAP31 may potentially interfere with STX17–ATG14 association through spatial competition.

We next experimentally evaluated the role of BAP31 under mechanical stress. Immunofluorescence staining showed that FFSS stimulation significantly increased BAP31 expression in chondrocytes, accompanied by enhanced colocalization between BAP31 and STX17. Pearson's correlation coefficient increased from r = 0.68 in control cells to r = 0.83 under FFSS conditions, indicating increased spatial association between these proteins (Figure 4C–E).

Consistently, Co‐IP assays showed that FFSS stimulation increased BAP31–STX17 interaction by 1.4‐fold (*p* < 0.001; Figure 4F–I). To further determine whether BAP31 directly interferes with ATG14 binding to STX17, we performed pull‐down assays in which STX17 and ATG14 levels were held constant while BAP31 was added at increasing concentrations. Increasing BAP31 levels progressively reduced the amount of ATG14 bound to STX17 (Figure 4J–L), indicating a dose‐dependent decrease in ATG14–STX17 association in the presence of BAP31. These findings suggest that BAP31 inhibits ATG14–STX17 association in a dose‐dependent manner, likely by affecting their binding interaction.

### 2.5 BAP31 Inhibition Rescues Autophagy Dysfunction and Suppresses Exosome Biogenesis

To assess whether BAP31 inhibition can alleviate autophagy impairment and reduce abnormal exosome release, chondrocytes were transfected with BAP31‐targeting small interfering RNA (siRNA) (Table S1) prior to FFSS stimulation. The knockdown efficiency was validated by WB analysis (Figure S9).

**FIGURE 5:**
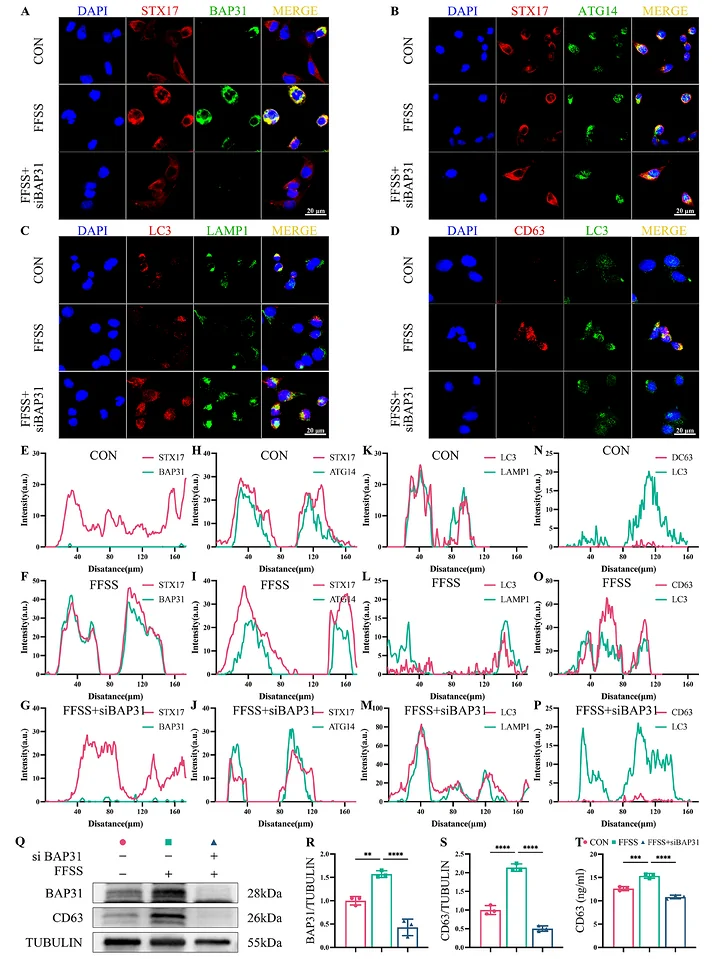
BAP31 inhibition rescues autophagy dysfunction and suppresses exosome biogenesis. (A–D) Representative IF images of (A) BAP31–STX17, (B) ATG14–STX17, (C) LC3–LAMP1, and (D) LC3–CD63 in treated ATDC5 cells. (E–P) Quantification of (E–G) BAP31–STX17, (H–J) ATG14–STX17, (K–M) LC3–LAMP1, and (N–P) LC3–CD63 staining in cultured chondrocytes under CON, FFSS, and FFSS + siBAP31 conditions. (Q) Representative Western blot analysis of BAP31 and CD63 protein expression in cells treated with CON, FFSS, or FFSS+siBAP31. (R,S) Quantitative analysis of (R) BAP31 and (S) CD63 expression levels across treatment groups (n = 3 biological replicates). (T) ELISA quantification of secreted CD63 in culture supernatants (n = 3). One‐way ANOVA. ^**^ *p* < 0.01, ^***^ *p* < 0.001, and ^****^ *p* < 0.0001.

Immunofluorescence analysis showed that FFSS markedly increased the colocalization between BAP31 and STX17, with Pearson's correlation coefficient rising from r = 0.47 in control cells to r = 0.92 under FFSS conditions. Notably, this association was significantly reduced following BAP31 knockdown (r = 0.20), indicating that siBAP31 effectively disrupts the spatial association between BAP31 and STX17 (Figure 5A,E–G). In contrast, the colocalization between ATG14 and STX17 was moderately reduced under FFSS stimulation (r = 0.66 in CON vs r = 0.57 in FFSS), but was markedly increased after BAP31 knockdown (r = 0.86), suggesting restoration of ATG14–STX17 association upon BAP31 inhibition (Figure 5B,H–J).

Consistently, LC3–LAMP1 colocalization was substantially decreased by FFSS (r = 0.65 in CON vs r = 0.18 in FFSS), indicating impaired autophagosome–lysosome association. This reduction was largely reversed by BAP31 knockdown (r = 0.63), suggesting improved autophagic processing (Figure 5C,K–M). In parallel, LC3–CD63 colocalization increased under FFSS stimulation (r = 0.10 in CON vs r = 0.56 in FFSS), indicating enhanced spatial association between autophagosomes and exosome‐related compartments. BAP31 knockdown markedly reduced LC3–CD63 colocalization (r = 0.19), suggesting decreased coupling between autophagosomes and exosome‐associated vesicular structures (Figure 5D,N–P). Western blot analysis confirmed that FFSS stimulation induced significant accumulation of LC3‐II and p62 in chondrocytes, whereas BAP31 knockdown significantly attenuated the FFSS‐triggered elevation of the LC3‐II/LC3‐I ratio and total p62 protein levels (Figure S5). Consistent with these findings, BAP31 knockdown significantly reduced CD63 levels both intracellularly (−66.3%; *p* < 0.001; Figure 5Q–S) and in the culture supernatant (−29.5%; *p* < 0.0001; Figure 5T), suggesting attenuation of exosome production.

To further verify that BAP31 regulates exosome secretion and downstream calcification through autophagic flux modulation, we performed rescue experiments by combining BAP31 knockdown with BafA1‐mediated autophagosome‐lysosome fusion blockade, and quantified CD63 levels in the culture supernatants by ELISA. FFSS stimulation significantly increased CD63 levels compared with the control group. In line with earlier findings, BafA1 treatment further elevated extracellular CD63 levels in FFSS‐stimulated chondrocytes, confirming that aggravated autophagic flux blockade promotes extracellular vesicle release. BAP31 knockdown consistently reduced CD63 secretion under both FFSS and FFSS+BafA1 conditions. Critically, the magnitude of CD63 reduction by siBAP31 was significantly attenuated in the presence of BafA1, indicating that the regulatory effect of BAP31 on extracellular vesicle release is partially dependent on autophagosome‐lysosome fusion. (Figure S10).

To further investigate the functional consequences of FFSS‐induced autophagic impairment, Alizarin Red S staining was performed to evaluate calcium deposition under different conditioned medium treatments. Compared with the control group, FFSS stimulation significantly increased calcium deposition in chondrocytes, indicating enhanced mineralization under mechanical stress. In the presence of BafA1, calcium deposition was further elevated in FFSS‐treated chondrocytes, suggesting that exacerbation of autophagic flux blockade promotes calcification. While BAP31 knockdown in donor cells significantly reduced calcium deposition in recipient chondrocytes under both FFSS and FFSS+BafA1 conditions, the protective effect of siBAP31 was partially abrogated when autophagosome‐lysosome fusion was blocked by BafA1. These findings indicate that BAP31‐mediated autophagic dysfunction contributes to FFSS‐induced chondrocyte calcification, at least in part (Figure S11).

In summary, these data suggest that inhibition of BAP31 restores the spatial association of STX17 and ATG14, rescues autophagosome–lysosome fusion, suppresses exosome biogenesis, and attenuates the pro‐calcific phenotype in vitro.

### 2.6 Chondrocyte‐Specific BAP31 Deletion Restores Autophagic Flux and Attenuates Pathological Calcification in OA

To investigate the therapeutic potential of BAP31 inhibition in vivo, we used a COL2A1‐targeted adeno‐associated virus serotype 2 (AAV2) system for chondrocyte‐specific delivery of short hairpin RNA against BAP31 (Table S2). Pilot studies characterizing viral kinetics revealed that intra‐articularly administered AAV2 carrying a ubiquitous promoter and EGFP induced initial chondrocyte transgene expression at 2 weeks, which peaked at 4 weeks and was largely diminished by 8 weeks (Figure S12). On the basis of this profile, COL2A1‐AAV2‐shBAP31 was administered at 2 weeks post‐UAC modeling, allowing robust transgene expression during the early to middle stages of OA progression before tissue collection at week 8. The BAP31 knockdown efficiency was validated by immunoblotting (Figure S13).

**FIGURE 6:**
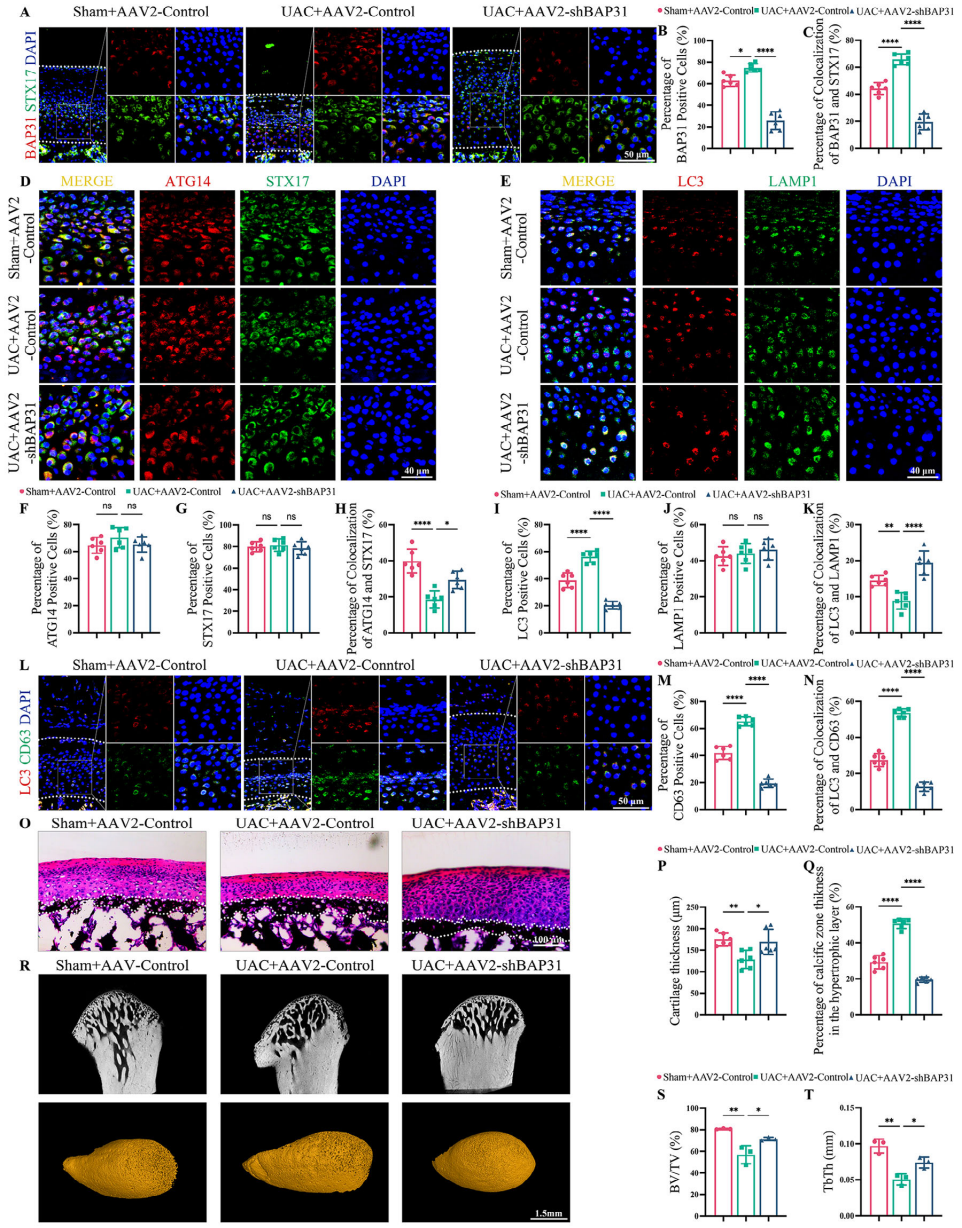
Chondrocyte‐specific BAP31 deletion restores autophagic flux and attenuates pathological calcification in OA. (A) Representative IF images of BAP31–STX17 in condylar cartilage. (B,C) Quantification of BAP31^+^ cell and BAP31–STX17 colocalization (n = 6 animals/group). (D) Representative IF images of ATG14–STX17 in condylar cartilage. (E) Representative IF images of LC3–LAMP1 in condylar cartilage. (F–K) Quantification of ATG14^+^ cells, STX17^+^ cells, ATG14–STX17 colocalization, LC3^+^ cells, LAMP1^+^ cells, and LC3–LAMP1 colocalization in different groups (n = 6). (L) Representative IF images of LC3–CD63 in condylar cartilage. (M,N) Quantification of CD63^+^ cells and LC3–CD63 colocalization across groups (n = 6). (O) Representative von Kossa staining images of condylar cartilage across groups. (P) Quantification of condylar cartilage thickness across groups (n = 6 animals per group). (Q) Quantification of the ratio of pathologically calcified zone to the entire hypertrophic layer in cartilage across groups (n = 6). (R) Representative micro‐CT reconstruction of condylar cartilage across groups. (S) Quantification of the BV/TV across groups (n = 3). (T) Quantification of trabecular thickness across groups (n = 3). One‐way ANOVA. ns, not significant; ^*^ *p* < 0.05, ^**^ *p* < 0.01, ^***^ *p* < 0.001, and ^****^ *p* < 0.0001.

COL2A1‐AAV2‐shBAP31 treatment at 8 weeks markedly suppressed BAP31 expression by 65.6% (*p* < 0.0001) and reduced BAP31–STX17 colocalization by 70.6% (*p* < 0.0001; Figure 6A–C). Crucially, this effect was accompanied by a 1.5‐fold increase in ATG14–STX17 colocalization (*p* < 0.05), restoring it to 73.7% of that in the Sham+AAV2‐Control group (*p* < 0.01; Figure 6D,F–H). Autophagic flux significantly recovered, with LC3–LAMP1 colocalization increasing 2.2‐fold compared with that in the UAC+AAV2‐Control group (*p* < 0.0001; Figure 6E,I–K). Furthermore, CD63 expression was reduced to 29.8% of that in the UAC+AAV2‐Control group (*p* < 0.0001), and LC3–CD63 colocalization decreased by 76.5% (*p* < 0.0001; Figure 6L–N), indicating suppression of exosome biogenesis.

We next evaluated whether BAP31 inhibition ameliorated pathological changes in vivo. H&E staining showed that BAP31 knockdown partially improved cartilage structure, as evidenced by better‐preserved tissue architecture and increased cartilage thickness compared with the UAC+AAV2‐Control group (1.3‐fold increase, *p* < 0.0001, Figure 6P; Figure S14). Histological evaluation by Safranin O staining further confirmed the protective effect of BAP31 silencing on cartilage matrix preservation. Compared with the UAC+AAV2‐Control group, COL2A1‐AAV2‐shBAP31‐treated joints exhibited increased proteoglycan retention and a significantly enlarged Safranin O‐positive area (2.5‐fold increase, *p* < 0.001), accompanied by a marked reduction in modified Mankin scores (*p* < 0.001; Figure S15). Immunohistochemical staining for type II collagen (Col II) and matrix metalloproteinase 13 (MMP13) further supported these findings, showing increased Col II expression and markedly reduced MMP13 levels following BAP31 knockdown (Figure S16).

Importantly, Von Kossa staining revealed that BAP31 inhibition reduced the proportion of calcified hypertrophic chondrocytes compared with the UAC group (*p* < 0.05; Figure 6O–Q), indicating attenuation of pathological calcification. In addition, micro‐CT analysis demonstrated improvements in subchondral bone microstructure, with increased bone volume fraction (BV/TV) and trabecular thickness (Tb.Th) in the BAP31‐inhibited condyles (*p* < 0.05; Figure 6R–T).

To determine whether elevated BAP31 expression is sufficient to induce pathological changes in vivo, chondrocyte‐specific BAP31 overexpression was established by intra‐articular injection of COL2A1‐AAV2‐BAP31. Compared with the Sham+AAV2‐Control group, BAP31 overexpression markedly increased BAP31–STX17 colocalization by 1.7‐fold (*p* < 0.0001) and reduced ATG14–STX17 colocalization by 56.2% (*p* < 0.001; Figure S17A,B,E–J). Autophagic flux was significantly impaired, as evidenced by a 44.5% decrease in LC3–LAMP1 colocalization (*p* < 0.001; Figure S17C,K–M). Furthermore, CD63 expression was elevated, and LC3–CD63 colocalization increased substantially (*p* < 0.0001; Figure S17D,N–P), indicating enhanced exosome biogenesis.

We next evaluated whether BAP31 overexpression aggravated pathological changes in vivo. Histological analyses demonstrated that BAP31‐overexpressing joints exhibited a 1.7‐fold increase in pathological calcification (*p* < 0.01), a 31.1% reduction in cartilage thickness (*p* < 0.001), significantly increased modified Mankin scores (*p* < 0.01), and a 68.9% decrease in the Safranin O‐positive area (*p* < 0.001) compared with the Sham+AAV2‐Control group (Figure S17Q–X). These findings indicate that elevated BAP31 expression alone is sufficient to induce OA‐like cartilage degeneration and pathological calcification in vivo.

Taken together, these bidirectional genetic manipulations demonstrate that chondrocyte‐specific BAP31 drives autophagic flux impairment, exosome overproduction and pathological cartilage calcification in OA, while targeted BAP31 inhibition restores autophagic homeostasis and attenuates cartilage degeneration.

## 3 Discussion

Pathological cartilage calcification in OA, including temporomandibular joint, is increasingly considered an important pathogenic feature contributing to the onset and progression of OA rather than a passive degenerative change [6], yet its mechanotransduction pathways remain poorly understood. While exosomes have been implicated in promoting calcification in cardiovascular contexts, which is often linked to dysregulated autophagy, their role in OA‐related calcification remains unclear [29]. Here, we report that BAP31 is a mechanosensitive regulator that couples mechanical stress to impaired autophagy and exosome‐mediated calcification. Specifically, we demonstrate that mechanical stress triggers BAP31 overexpression in chondrocytes, which competitively disrupts STX17–ATG14‐mediated autophagosome‒lysosome fusion. This disruption redirects accumulated autophagosomes toward the generation of calcification‐competent exosomes. Importantly, targeted inhibition of BAP31 in a mechanically induced OA (UAC) model effectively alleviated pathological calcification in cartilage and attenuated disease progression, highlighting its therapeutic potential.

Emerging evidence further indicates that chondrocyte‐derived exosomes act as functional mediators linking intracellular stress responses to extracellular matrix remodeling. These vesicles are not merely byproducts but active regulators of cartilage calcification and may contribute to disease propagation within the joint microenvironment [18]. For instance, Shan et al. reported that exosomes from melatonin‐treated endothelial cells alleviate calcification in vascular smooth muscle cells by activating the miR‐302d‐5p/Wnt3 signaling pathway in an m6A methylation‐dependent manner [30]. Conversely, Liu et al. reported that nanoscale hydroxyapatite (nHAp) disrupts lysosomal acidification, impairing autophagy, promoting the conversion of accumulated autophagosomes into exosomes and leading to the secretion of exosomes loaded with calcifying factors [29]. Consistent with these findings, autophagy inhibitors have been shown to significantly exacerbate cartilage degeneration in OA [31]. Similarly, our in vivo and in vitro experiments confirmed that exosome depletion counteracts mechanical stress‐induced calcification.

Despite this growing body of evidence, the upstream signals that drive the increased release of pro‑calcific exosomes under pathological mechanical loading remain largely undefined. A compelling body of work points to autophagic dysfunction as a central node: impaired autophagic flux reroutes multivesicular bodies from lysosomal degradation toward plasma membrane fusion, thereby enhancing exosome secretion and altering their cargo composition [32, 33]. However, its upstream regulators exhibit tissue specificity. For instance, Gao et al. linked defective lysosomal acidification due to V‐ATP6V1A deficiency to autophagosome accumulation and ectopic calcification in tendons [34]. In the context of OA, upregulation of histone deacetylase 6 (HDAC6) expression has been shown to disrupt microtubule‐dependent autophagosome trafficking, leading to excessive release of LC3^+^ exosomes and subsequent cartilage calcification [20, 35]. Importantly, modulation of autophagic activity in that study directly altered extracellular vesicle release and calcification phenotypes, providing functional evidence that autophagy‐dependent EV biogenesis is not merely correlative but causally linked to matrix mineralization [20]. In our study, we revealed a novel pathway through which mechanical stress triggers the overexpression of BAP31, an endoplasmic reticulum‐resident membrane protein [36, 37], which competitively displaces ATG14 from the STX17 binding interface. This molecular interference reduces ATG14–STX17 colocalization by 70.5%, thereby disrupting autophagosome‒lysosome fusion and redirecting accumulated autophagosomes. Consequently, undegraded autophagosomes promote plasma membrane budding, forming calcification‐competent vesicles that couple mechanotransduction to matrix calcification.

Although BAP31 is widely expressed and has been implicated in diseases ranging from cancer [38, 39] to neurological disorders [24, 40, 41], its role in OA has not previously been established. Notably, while Namba et al. reported that BAP31 depletion enhances autophagosome formation via AMPK‐ULK1 activation [42], our data reveal an additional role in autophagosome‒lysosome fusion impairment, resulting in pronounced autophagosome accumulation. Therapeutically, chondrocyte‐specific knockdown of BAP31 elicited a multilevel restorative response: molecularly, the ATG14–STX17 complex was restored to 73.7% of the Sham+AAV2‐Control group levels; cellularly, autophagic flux recovered, as evidenced by a 2.2‐fold increase in LC3–LAMP1 colocalization; and tissue‐level pathological calcification was attenuated, with a 61.4% reduction in calcification. This tripartite rescue effect spans molecular interactions, organelle dynamics, and tissue pathology, validating BAP31 as a therapeutically actionable node. Notably, intra‐articular AAV2‐mediated knockdown resulted in functional recovery without altering physiological calcification, suggesting a favorable therapeutic window for targeting this pathway in mechanically stressed joints.

While this study delineates the role of the BAP31–STX17 axis in the mechanical stress response, several limitations should be acknowledged. First, the upstream mechanosensor regulating BAP31 expression remains uncharacterized; single‐cell transcriptomics of mechanically stimulated chondrocytes may help identify potential upstream regulators. Second, although TEM and colocalization analyses support exosome biogenesis from accumulated autophagosomes, direct dynamic evidence remains limited. In particular, live‐cell imaging of LC3^+^ vesicle secretion or the use of an mRFP–GFP–LC3 reporter system would provide more direct and real‐time assessment of autophagic flux and vesicle trafficking. Third, proteomic profiling of exosomal cargo remains incomplete; further characterization of calcification‐related molecules, such as ANKH and ENPP1, is warranted to identify key mediators of extracellular matrix mineralization. Finally, although micro‐CT analysis suggested that BAP31 intervention restored subchondral bone microarchitecture, including trabecular thickness and bone volume fraction, to levels comparable to the Sham+AAV2‐Control group, we did not directly assess osteogenic activity or molecular markers of subchondral bone remodeling. Therefore, the potential role of exosome‐mediated signaling in regulating subchondral bone remains unclear and requires further investigation.

In conclusion, our study provides mechanistic insights into OA‐associated cartilage calcification and suggests that BAP31 inhibition is a promising strategy for mechanically driven OA. Notably, these observations do not preclude the potential physiological roles of BAP31 upregulation in other contexts. Future therapeutic developments should therefore carefully consider joint homeostasis and seek to balance BAP31 overexpression with the prevention of pathological calcification processes.

## 4 Experimental Methods

### 4.1 Animals

Sprague–Dawley (SD) rats (female, 6 weeks old, 140–160 g) were obtained from the Air Force Medical University Experimental Animal Center. All experimental protocols were approved by the institutional ethics committee (No. kq‐2024‐026) and conducted in accordance with the ARRIVE guidelines, adhering to the 3R principles (Replace, Reduce, Refine) to ensure humane treatment of the animals.

### 4.2 Uac Model

Metal sleeves were fabricated using standardized techniques prior to experimentation. For maxillary applications, 25‐gauge mammary duct probes were precision‐formed into 2–3 mm cylindrical metal sleeves. Mandibular prostheses were constructed from 20‐gauge mammary duct probes, with one aspect mechanically flattened to create an anatomical guide plate (3.5–4 mm in length) oriented at a consistent 45° angle relative to the prosthetic axis while maintaining a 3 mm sleeve segment. All experimental procedures were performed under standardized conditions. SD rats in the experimental cohort were anesthetized using isoflurane administered through a calibrated rodent ventilator system. Following operative field isolation, zinc phosphate cement was prepared at the optimal viscosity for prosthesis fixation (string‐stage consistency). Sham control animals received identical anesthetic and preparatory treatments, excluding prosthesis placement. All surgical interventions were consistently completed within a 5‐min window to minimize procedural variability. Prosthesis retention was maintained throughout the experimental period. In the event of dislodgement, a new prosthesis was immediately reattached. All the animals were maintained in controlled, identical housing environments with ad libitum access to food and water. The rats were euthanized at the predetermined experimental endpoint. Transcardial perfusion fixation was performed within 5 min after death was confirmed. Mandibular condylar cartilage specimens were subsequently dissected for further analysis.

### 4.3 Intra‐Articular Drug Injection

Following isoflurane anesthesia, bilateral injections of the adeno‐associated virus (AAV) were administered into the temporomandibular joints of SD rats using 1 mL insulin syringes (29G needles). The injection site was precisely located at the middle‐posterior junction of the zygomatic arch (approximately 5 mm anterior to the tragus), with the needle inserted at a standardized 45° anteromedial‐superior trajectory to a depth of 0.5–0.9 cm. After aspiration was confirmed negative for blood, the corresponding AAV constructs were slowly infused into each joint (5.3×10^1^ ^2^ vg mL^−1^, 50 µL per joint). The experimental groups (AAV2‐BAP31 group and UAC+AAV2‐shBAP31 group) received the corresponding AAV constructs, while the sham and UAC‐only groups received the same volume and titer of the control AAV (AAV2‐Control) with identical injection techniques. Mandibular condyle specimens were systematically harvested at the predetermined 8‐week endpoint for subsequent analysis.

### 4.4 Cell Culture

ATDC5 cells were cultured in complete medium (DMEM supplemented with 10% fetal bovine serum and 1% penicillin‒streptomycin) at 37°C and 5% CO_2_. The medium was changed and the cells were subcultured every 1–2 days depending on cell confluence and growth status for subsequent experiments.

### 4.5 Construction of the Cellular Fluid Shear Stress Model

ATDC5 chondrogenic cells at 90% confluence were enzymatically dissociated using 0.25% trypsin‐EDTA (37°C, 3 min) after being washed twice with PBS. Trypsinization was stopped with complete growth medium. After centrifugation (1,500 r min^−1^, 3 min), the cell pellet was resuspended in fresh complete medium and seeded at a density of 10^6^ cells per cm^2^ on Streamer Culture Slips (1 mm thickness, CS‐C/FFCS‐C, USA) precoated with Collagen I. After 24 h of attachment, the experimental groups were subjected to laminar fluid shear stress (24 dyn per cm^2^, 2 h) using a calibrated flow system (Streamer Fluid Shear Stress Device), while the control groups were maintained under static conditions. All the cultures were subsequently incubated for 6 h (37°C, 5% CO_2_) prior to analysis.

### 4.6 Cell Transfection

ATDC5 chondrocytes were cultured in 6‐well plates to 70–80% confluence prior to transfection. The transfection complexes were prepared as follows: Solution A: 8 µl of GP‐Transfect‐Mate transfection reagent (GenePharma) was gently mixed with 200 µl of serum‐free DMEM in a sterile 1.5 mL microcentrifuge tube and incubated at room temperature for 5 min; Solution B: 150 pmol of either siRNA against BAP31 or negative control siRNA (GenePharma) was diluted in 200 µl of serum‐free DMEM and similarly incubated. The transfection mixture was prepared by gradually adding Solution A to Solution B while gently pipetting and then incubated at room temperature for 15–20 min. Next, the cells were washed twice with PBS (pH 7.4) and maintained in 1,600 µl of serum‐free DMEM. The transfection complexes (400 µl) were then added dropwise to each well (the final volume was 2 mL). After 4–6 h of incubation at 37°C and 5% CO_2_, the medium was replaced with complete growth medium. Cells were processed for either protein extraction or fluid shear stress experiments at 24 h post transfection.

### 4.7 Molecular Docking Analysis

Protein sequence and structure data were obtained by querying the UniProt repository (https://www.uniprot.org/uniprotkb). The retrieved structures were subsequently imported into Discovery Studio 2019 to undergo a series of optimization procedures. These procedures included the removal of extraneous water molecules, the addition of hydrogen atoms, the assignment of appropriate charges, the completion of any missing amino acids, and the rebuilding of incomplete side chains. Following optimization, the refined protein structures were imported into the GRAMM software for protein‒protein docking simulations. The docking outcomes were then visualized using PyMOL 3.1 to facilitate detailed analysis and interpretation.

### 4.8 Statistical Analysis

The experimental data were analyzed using GraphPad Prism 10.0 (GraphPad Software Inc., San Diego, CA, USA). Quantitative results are presented as mean ± standard deviation (SD), and the sample size (n) for each statistical analysis is indicated in the corresponding figure legends. The assumptions of normality and homoscedasticity were verified using the Shapiro‒Wilk test and Levene's test, respectively. Differences between two groups were evaluated using the unpaired Student's t test, and differences among three or more groups were evaluated using one‐way analysis of variance (ANOVA), followed by the Holm‒Šidák post hoc test for multiple comparisons. All statistical tests were two‐sided. A p value of less than 0.05 was considered to indicate statistical significance.

### 4.9 Reagents and Protocols

Key reagents and experimental parameters are listed in Table S3. The detailed methods are described in the Supporting Information.

## Author Contributions

Conceptualization: Mian Zhang, Feng He. Experiments: Zhihua Xu, Fuyin Li, Yu Jiang, Qinghua Li. Data analysis: Zhihua Xu, Ruoxin Wang, Fuyin Li, Min Hui, Yuqian Shi. Project administration and supervision: Mian Zhang. Funding acquisition: Mian Zhang, Qian Liu, Qiannan Niu, Feng He, Shibin Yu. Writing the manuscript: Zhihua Xu. Reviewing the manuscript: Mian Zhang, Feng He, Junwen Jing, Litian Ma.

## Conflicts of Interest

The authors declare no conflicts of interest.

## Ethics Statement

Ethics approval was obtained from the Ethics Committee of The Fourth Military Medical University.

## Supporting information




**Supporting File 1**: advs77405‐sup‐0001‐SuppMat.docx.

**Supporting File 2**: advs77405‐sup‐0002‐Data.zip.

**Supporting File 3**: advs77405‐sup‐0003‐Table S1.xlsx.

**Supporting File 4**: advs77405‐sup‐0004‐Table S2.xlsx.

**Supporting File 5**: advs77405‐sup‐0005‐Table S3.xlsx.

## Data Availability

The data that support the findings of this study are available from the corresponding author upon reasonable request.
