# Supplementary material for: BAP31 Drives Cartilage Calcification through Disruption of Autophagosome–Lysosome Fusion in Osteoarthritis
Source: Adv Sci (Weinh). 2026 Aug 29:e20140. Online ahead of print. doi: 10.1002/advs.202520140 (PMC13525426; doi:10.1002/advs.202520140)
Supplement: Supplementary file 1 [file ADVS-9999-e20140-s004.docx]

**Supporting Information**

**BAP31 Drives Cartilage Calcification through Disruption of Autophagosome–Lysosome Fusion in Osteoarthritis**

Zhihua Xu^#1^, Ruoxin Wang^#2^, Feng He^#3^, Qian Liu^#4^, Fuyin Li^#1^, Min Hui^5^, Yuqian Shi^1^, Yu Jiang^6^, Qinghua Li^1^, Junwen Jing^1^, Shibin Yu^1^, Litian Ma*^7,8^, Qiannan Niu*^2^, Mian Zhang*^1^

**Figure S1** Representative H&E staining images of condylar cartilage in the Sham, Sham+GW4869, UAC, and UAC+GW4869 groups.

**Figure S2** Expression of CD63 in ATDC5 cells with or without FFSS stimulation.

A. CD63 expression in ATDC5 cells with or without FFSS stimulation determined by western blotting. B. Quantification of CD63 protein levels in different groups (n=3 independent experiments).

**Figure S3** A. Representative TEM image of exosomes from the control group. Scale bar, 50 nm. B. Elemental analysis of exosome in (A).

**Figure S4** Representative Alizarin Red S staining images of chondrocytes from different treatment groups.

**Figure S5** BafA1-induced LC3-II, p62 and LAMP1 accumulation in different groups.

A. Representative immunoblot images of LC3-II, p62, and LAMP1 expression in chondrocytes from different groups following BafA1 treatment. B. Quantification of the LC3-II/LC3-I ratio in each group. (n=3 biological replicates). C. Quantification of p62 protein levels in chondrocytes from different groups following BafA1 treatment (n=3). D. Quantification of LAMP1 protein levels in chondrocytes from different groups following BafA1 treatment (n=3). One‐way ANOVA. ns not significant, *p<0.05, **p<0.01, ***p<0.001 ****p<0.0001.

**Figure S6** Relative LC3B-II/CD63 ratio in CON-exos and FFSS-exos based on Western blot results.

**Figure S7** Expression of ATG14 and STX17 in chondrocytes from different groups

A. Quantification of ATG14-positive chondrocytes in different groups (n=3). B. Quantification of STX17-positive chondrocytes in different groups (n=3). Unpaired Student’s *t* test. ns not significant.

**Figure S8** Top five STX17 interactors ranked by evidence strength.

**Figure S9** Validation of BAP31 knockdown efficiency in ATDC5 cells.

A. Western blot analysis of BAP31 protein expression. B. Statistical analysis of BAP31 protein levels (n=3 biological replicates; ns P>0.05, **P<0.01).

**Figure S10** BAP31 regulates FFSS-induced exosomal CD63 secretion.

**Figure S11** Representative Alizarin Red S staining images of chondrocytes from different treatment groups.

**Figure S12** AAV transduction efficiency and persistence following intra-articular injection.

A. Fluorescence imaging of AAV-mediated gene expression in joint tissues. (AAV2, 5.30×10^12^ vg ml^-1^; weeks 2, 4, and 8 postinjection).

**Figure S13** Validation of BAP31 knockdown efficiency in primary rat condylar chondrocytes.

A. Representative western blot images of BAP31 expression. B. Quantitative analysis of BAP31 protein levels (n=3 biological replicates; **P<0.01, ***P<0.001).

**Figure S14** Representative H&E staining images of condylar cartilage from different treatment groups.

**Figure S15** Chondrocyte-specific BAP31 silencing preserves cartilage matrix integrity and reduces OA severity.

A. Representative images of H&E staining of articular cartilage from Sham+AAV2-Control, UAC+AAV2-Control, and UAC+AAV2-shBAP31 groups. B. Representative Safranin O staining images showing proteoglycan content and cartilage matrix preservation among the three groups. C. Quantitative analysis of cartilage thickness across groups (n=6 animals per group). D. Quantitative analysis of Safranin O-positive staining area across groups (n=6). E. Quantitative analysis of modified Mankin score across groups (n=6). Data are presented as mean ± SD. Statistical significance was determined by one-way ANOVA followed by Tukey’s multiple comparisons test. *P < 0.05, **P < 0.01, ***P < 0.001.

**Figure S16** Chondrocyte-specific BAP31 silencing alleviates extracellular matrix degradation in articular cartilage.

A. Representative immunohistochemical staining of MMP13 in the articular cartilage of Sham+AAV2-Control, UAC+AAV2-Control, and UAC+AAV2-shBAP31 groups. B. Quantification of the percentage of MMP13-positive cells in the articular cartilage (n=6 animals per group). C. Representative immunohistochemical staining of COL-II in the articular cartilage of Sham, UAC+AAV2-Control, and UAC+AAV2-shBAP31 groups. D. Quantification of the COL-II positive area in the articular cartilage (n = 6). Statistical significance was determined by one-way ANOVA followed by Tukey's multiple comparisons test. *P < 0.05, **P < 0.01.

**Figure S17** BAP31 overexpression is sufficient to impair autophagic flux and exacerbate pathological calcification of articular cartilage in OA.

A. Representative IF images of BAP31–STX17 in condylar cartilage treated with AAV2-BAP31. B. Representative IF images of ATG14–STX17 in condylar cartilage treated with AAV2-BAP31. C. Representative IF images of LC3–LAMP1 in condylar cartilage treated with AAV2-BAP31. D Representative IF images of LC3–CD63 in condylar cartilage treated with AAV2-BAP31. E–G Quantitative analysis of BAP31^+^ cells (E), STX17^+^ cells (F), and the percentage of BAP31–STX17 colocalization (G). H–J Quantification of ATG14^+^ cells (H), STX17^+^ cells (I), and ATG14–STX17 colocalization (J). K–M Quantification of LC3^+^ cells (K), LAMP1^+^ cells (L), and LC3–LAMP1 colocalization (M). N–P Quantification of LC3^+^ cells (N), CD63^+^ cells (O), and LC3–CD63 colocalization (P). Q. Representative Von Kossa staining of condylar cartilage treated with AAV2-BAP31. R. Representative H&E staining of condylar cartilage treated with AAV2-BAP31. S Quantification of the ratio of pathologically calcified zone to the entire hypertrophic layer in cartilage across groups (n=6 animals per group). T. Quantification of condylar cartilage thickness across groups (n=6) U. Representative H&E staining of condylar cartilage treated with AAV2-BAP31. V. Representative Safranin O staining of condylar cartilage treated with AAV2-BAP31. W. Quantification of the modified Mankin score (n=6). X Quantification of the Safranin O-positive stained area (n=6). Statistical analyses were performed using an unpaired two-tailed Student's t-test. ns, not significant; ** P < 0.01; ***P < 0.001; ****P < 0.0001.

**Table S1.** siRNA sequences for BAP31 knockdown in mouse

| target gene | Primer sequences (5’-3’) |
| --- | --- |
| BAP31-mus-202 | F: GGAGUUGGUAGUGACCUAUTT  R: AUAGGUCACUACCAACUCCTT |
| BAP31-mus-375 | F: CUCAGAGGAAUCUCUAUAUTT  R: AUAUAGAGAUUCCUCUGAGTT |
| BAP31-mus-472 | F: CUCCAAUGAAGCCUUUAAATT  R: UUUAAAGGCUUCAUUGGAGTT |
| BAP31-mus-729 | F: GCCUUACCAAAGAAUAUGATT  R: UCAUAUUCUUUGGUAAGGCTT |
| Negative control | F: UUCUCCGAACGUGUCACGUTT  R: ACGUGACACGUUCGGAGAATT |

**Table S2.** Primer sequences for validation of BAP31 knockdown efficiency in rats

| target gene | Primer sequences (5’-3’) |
| --- | --- |
| BAP31-rat-si1 | F: CCGGAAAUAUGAUGAUGUATT  R: UACAUCAUCAUAUUUCCGGTT |
| BAP31-rat-si2 | F: GGAUGUUGGGAGUCCUGAATT  R: UUCAGGACUCCCAACAUCCTT |
| BAP31-rat-si3 | F: GUUGGUCAUUGAUGCUGUATT  R: UACAGCAUCAAUGACCAACTT |
| BAP31-rat-Primer | F: TCAGCTAAAGAAGGGAACTGCT  R: GCTTCTTCAGATCAGTCTTCAGG |
| Negative control | F: UUCUCCGAACGUGUCACGUTT  R: ACGUGACACGUUCGGAGAATT |

**Table S3.** Key reagents and experimental parameters

| Category | Reagent details | Concentration |
| --- | --- | --- |
| BAP31 | 11200-1-AP, Proteintech, China | 1:20000 for WB, 1:200 for IF (cell and frozen section) |
| STX17 | 17815-1-AP, Proteintech, China | 0.5μg for IP |
| STX17 | ab229646, abcam, USA | 1:200 for IF (cell frozen section), 1:5000 for WB |
| STX17-FITC | Bs-17151R-FITC, Bioss, China | 1:100 for IF (frozen section) |
| ATG14 | ab227849, abcam, USA | 1:200 for IF (cell) |
| P-PERK | DF7576, Affinity, USA | 1:1500 for WB, 1:200 for IF (frozen section) |
| PERK | 24390-1-AP, Proteintech, China | 1:3000 for WB, 1:200 for IF (frozen section) |
| MMP13 | 18165-1-AP, Proteintech, China | 1:2000 for WB |
| CD63 | Sc-5257, SANTA CRUZ, USA | 1:1000 for WB, 1:100 for IF (cell and paraffin section) |
| LC3B | 83506S, Cell Signaling, USA | 1:100 for IF (cell and paraffin section) |
| LC3A/B | #12741, Cell Signaling, USA | 1:100 for IF (cell and paraffin section) |
| LAMP1 | Ab208943, abcam, USA | 1:100 for IF (cell and paraffin section) |
| TUBULIN | T0023, Affinity, USA | 1:10000 for WB |
| Rabbit IgG | ab172730, abcam, USA | 5μg for IP |
| NP-40 Lysis Buffer | P0013F, Beyotime, China | 1% NP-40 |
| Goat anti-rabbit recombinant secondary antibody | RGAR001, Proteintech, China | 1:10000 for WB |
| Goat anti mouse recombinant secondary antibody | SA00001-1, Proteintech, China | 1:2000 for WB |
| Alizarin Red S Solution | G1450, Solarbio, China | 0.2%, pH8.3 |
| FlexAble CoraLite Plus 594 Antibody Labeling Kit for Rabbit IgG | KFA009, Proteintech, China | refer to the manufacturer's instructions |
| FlexAble CoraLite Plus 488 Antibody Labeling Kit for Rabbit IgG | KFA001, Proteintech, China | refer to the manufacturer's instructions |
| Goat Anti-Mouse IgG H&L(Alexa Fluor 594) preadsorbed | ab150120, abcam, USA | 1:500 for IF |
| Goat Anti-Rabbit IgG H&L(Alexa Fluor 594) | ab150080, abcam, USA | 1:500 for IF |
| Goat Anti-Rabbit IgG H&L(Alexa Fluor 488) | Ab150077, abcam, USA | 1:500 for IF |
| Alexa Fluor 488-conjugatedAffiniPure Fab Fragment GoatAnti-Rabbit lgG (H+L) | 111-5475003, Jackson, USA | 1:200 for IF |
| Veriblot for IP Detection Reagent (HRP) | Ab131366, abcam, USA | 1:4000 for WB |
| GW4869 | S7609, Selleck, USA | 10 μg μl^-1^, with 50 μl injected into each joint. |
| Mouse CD63 ELISA Kit | MK0331MB, MEIKE, China | 48 T |
| Bafilomycin A1 | T6740, TargetMol, USA | 3nM |
| rapamycin | S1039, Selleck, USA | 100nM |

**Experimental Methods**

**Von Kossa staining**

The collected samples were immediately fixed in 4% paraformaldehyde (24–48 h, 4°C) followed by cryoprotection in 30% sucrose solution at 4°C until tissue sedimentation was complete. After surface moisture was carefully removed using sterile filter paper, the condylar tissues were embedded in optimal cutting temperature compound (4583; Sakura Tissue-Tek, USA) and snap-frozen in liquid nitrogen-cooled isopentane prior to storage at -80°C. Serial sagittal sections (10 μm thickness) were obtained using a cryostat (HM525NX; Thermo Scientific, USA) maintained at -20°C.

For histological evaluation, cryosections underwent sequential processing: (1) PBS rehydration (5 min, orbital shaking at 100 rpm), (2) 1% silver nitrate impregnation (30 min UV exposure at 365 nm), (3) 5% sodium thiosulfate treatment (5 min), (4) hematoxylin‒eosin staining with precise differentiation (hematoxylin: 5 min; acid alcohol: 1 s; eosin: 30 s), and (5) graded ethanol dehydration (70%, 95%, 100% ×2, 5 min each). Processed sections were coverslipped using Neutral quick-drying mounting medium (241011; Jiangyuan, China) and examined under a Leica DM2500 research-grade inverted microscope equipped with a high-resolution digital camera (DFC450 C; Leica). Von Kossa-positive staining was quantified as the ratio of pathologically calcified zone to the entire hypertrophic layer in cartilage. All histomorphometric analyses were performed using ImageJ software (NIH, 2.14.0/1.54f) with standardized calibration protocols.

**Hematoxylin and eosin (H&E) staining**

The preparation of frozen sections was performed as described in Section Von Kossa staining of this part.

Hematoxylin and Eosin staining (H&E staining) was performed on independent sections derived from the same frozen TMJ tissue blocks. Although consecutive sectioning was not feasible due to the technical limitations of undecalcified hard-tissue frozen preparation, strict anatomical matching and standardized region-of-interest (ROI) selection criteria were applied. Specifically, sections were selected from the central weight-bearing region of the mandibular condylar cartilage, and imaging was consistently focused on the mid-to-deep hypertrophic cartilage zone across all groups to ensure comparability.

H&E staining of frozen sections was carried out sequentially as follows: (1) PBS washing for 5 min at 100 rpm; (2) hematoxylin staining for 5 min, followed by rinsing with running water; (3) differentiation in acid alcohol for 1 s, followed by rinsing with running water; (4) eosin counterstaining for 30 s, followed by rinsing with running water; and (5) dehydration in absolute ethanol for 5 min. After staining, the sections were mounted with Neutral quick drying mounting medium and observed under a Leica DM2500 optical microscope for image acquisition. All histomorphometric analyses were performed using ImageJ software with standardized calibration protocols. All quantitative measurements were performed independently by two researchers in a blinded manner to minimize observer bias. For each animal, predefined ROIs on multiple valid sections were analyzed, and the mean value per animal was used for statistical analysis, with 6 biological replicates per group.

**Immunofluorescence (IF) staining**

Tissue section processing

Cryosections

After equilibration to room temperature, the sections (10 μm) were rinsed in PBS (5 min).

Paraffin-embedded sections

Sections (5 μm) were dried overnight at 60°C, deparaffinized in xylene (2 × 30 min), and rehydrated through a graded ethanol series (100%, 95%, 85%, and 75%; 5 min each) followed by distilled water (5 min). Antigen retrieval was performed in preheated citrate buffer (pH 6.0, 80°C, 10 min), after which the samples were cooled to room temperature. The sections were subsequently washed three times in PBS (pH 7.4, 5 min each).

Cell preparation

Cells were fixed with 4% paraformaldehyde at 4°C for 30 min, permeabilized with Triton X-100 (0.1% in PBS, 10 min, RT), and washed three times with PBS (5 min each).

Nonspecifically bound sites were blocked with 10% normal goat serum (2 h, RT). After serum removal, primary antibodies (optimized concentrations on the basis of the manufacturer's instructions or preliminary experiments) were applied, and the sections were incubated in a humidified chamber at 4°C overnight. The following day, the sections were warmed to room temperature (30 min) and subjected to four 5-min washes with PBS. All subsequent steps were performed under light-protected conditions. Fluorescent-conjugated secondary antibodies (1:500 dilution with DAPI) were applied (1 h, RT), followed by four additional 5-min washes with PBS. After the excess liquid was carefully removed, the sections were mounted with antifade mounting medium and sealed with clear nail polish. Imaging was performed using a laser scanning confocal microscope (FV1000; Olympus, Tokyo, Japan) with standardized acquisition parameters, including laser power, gain, offset, and exposure time, to ensure signal comparability and avoid saturation artifacts.

For quantitative analysis, images were acquired using full-field imaging within predefined regions of each sample rather than selecting individual cells. To ensure representativeness and minimize sampling bias, a minimum of five randomly selected fields per sample were analyzed under identical imaging conditions. For figure presentation, only representative cropped regions were displayed for visual clarity. Co-localization between fluorescence signals was quantified using Pearson’s correlation coefficient (PCC) at the pixel intensity level, providing an objective and standardized assessment of spatial correlation between markers. Image analysis was performed using a consistent thresholding and processing pipeline across all samples. All analyses were conducted in a blinded manner. Overexposed or saturated images were excluded from analysis to prevent artificial signal aggregation and ensure the reliability of quantitative measurements.

**Exosome isolation**

ATDC5 cells were uniformly seeded on Streamer™ Culture Slips at a density of 10^6^ cells per cm². After the application of FFSS, ATDC5 cells were cultured in complete medium supplemented with EV-depleted FBS. After 24 hours, the supernatants of the control and FFSS groups were collected separately. The supernatants were first filtered through a syringe filter and then sequentially centrifuged at 300 × g for 5 min, 2,000 × g for 10 min, and 10,000 × g for 30 min at 4°C to remove cells and debris, with the pellets discarded after each centrifugation. The supernatant was then filtered through a 0.45 μm filter membrane and subjected to ultracentrifugation at 100,000 × g for 70 min at 4°C (WX100, Thermo, USA). The supernatant was discarded, and the pellet was resuspended in PBS and filtered through a 0.22 μm filter membrane. The filtrate was collected and subjected to ultracentrifugation at 100,000 × g for 70 min at 4°C (WX100, Thermo, USA). The final pellet containing the exosomes was resuspended in PBS. At the designated collection time, the percentage of viable cells was more than 98%.

**Transmission electron microscopy (TEM) and elemental mapping**

Twenty microliters of the resuspended exosome samples were added dropwise to 200-mesh grids and incubated at room temperature for 10 min, after which the grids were negatively stained with 2% phosphotungstic acid (22400; EMS, USA) for 3 min and the remaining liquid was removed with filter paper. Transmission electron microscopy (TEM) images were acquired using a JEOL JEM1400 Plus microscope (JEOL, Tokyo, Japan) operated at 120 kV. For elemental mapping, energy-dispersive X-ray spectroscopy (EDS) was performed using an EDS system (JED-2300T, JEOL, Tokyo, Japan) attached to the TEM (JEOL F200, JEOL, Tokyo, Japan). The elemental distribution within the exosomes was analyzed and mapped using an EDS system.

**Alizarin red staining**

After exposure to FFSS, the cells were cultured for 6 h. Supernatants were harvested from both control and FFSS-treated cultures and divided into three cohorts: untreated control supernatant (CON-CM), FFSS-stimulated supernatant (FFSS-CM), and FFSS-CM following exosome depletion (FFSS-CM^-exos^). Cells were cultured with CON-CM, FFSS-CM, or FFSS-CM^-exos^ for 3, 7, 14, or 21 days. The culture medium was changed every 48 h throughout the experimental period.

For staining, the cells were washed twice with PBS, fixed in 4% paraformaldehyde for 10 min, and then rinsed twice with DEPC-treated water. Alizarin red staining solution (G1450; Solarbio, pH 8.3) was added, followed by incubation at 37°C for 30 min. After the samples were washed twice with absolute ethanol, they were visualized and imaged using an optical microscope (Nikon, Japan).

**Western blot**

Cell lysis was performed on ice using RIPA buffer supplemented with phosphatase and protease inhibitors (100:1). The lysates were subsequently centrifuged at 12,000 × g for 10 min at 4°C, after which the resulting supernatants were transferred to fresh sterile tubes. The samples were mixed with 5× loading buffer (1:4 ratio) and denatured at 100°C for 10 min using a constant-temperature metal bath shaker.

Protein samples were separated by SDS‒PAGE at 150 V constant voltage, followed by electrophoretic transfer to PVDF membranes at 1 A (20 V) for 30 min. The membranes were briefly rinsed with distilled water (1 min), blocked with 5% BSA in TBST for 2 h at room temperature, and then washed once with TBST (5 min). Primary antibody incubation was performed overnight at 4°C, followed by three TBST washes (5 min each). The membranes were then incubated with HRP-conjugated secondary antibodies for 1 h at room temperature and washed three times with TBST (5 min each). Protein bands were visualized using enhanced chemiluminescence substrate, and quantitative analysis was performed by densitometry using ImageJ software.

**Sample preparation for Coimmunoprecipitation (co-IP)**

Immunoprecipitation was performed using magnetic bead-based purification. Briefly, 60 μl of protein A/G magnetic beads were aliquoted into sterile microcentrifuge tubes and immobilized on a magnetic rack to remove the storage solution. The beads were washed three times with 1 ml of ice-cold RIPA lysis buffer (10 min each at 4°C with gentle rotation). Antibody coupling was achieved by incubating 5 μg of specific antibodies with the beads overnight at 4°C with rotation. Following three additional RIPA washes under identical conditions, the beads were resuspended in 1 ml of IP lysis buffer.

For sample preparation, the cells were lysed in IP buffer supplemented with protease and phosphatase inhibitors (1:100) on ice. After centrifugation (12,000 × g, 10 min, 4°C), the cleared lysates were incubated with antibody-conjugated beads overnight at 4°C. The immunocomplexes were then washed four times with RIPA buffer (10 min each at 4°C) and eluted in 30 μl of 2× loading buffer by boiling at 100°C for 10 min.

**Glutathione S-transferase (GST) pull-down assays**

For GST pull-down assays, the following recombinant proteins were expressed and purified from E. coli:

GST-STX17: The coding sequence corresponding to mouse syntaxin-17 (STX17, amino acid residues 1–227, UniProt Q9D014) was cloned into the pGEX-6P-1 vector. The fusion protein contains an N-terminal GST tag and has a predicted molecular mass of approximately 51 kDa (including the tag).

His-ATG14: The full-length coding sequence of mouse autophagy-related 14 (ATG14, residues 1–492, UniProt Q8CDJ3) was cloned into the pET-SUMO vector. The fusion protein carries an N-terminal His6 tag and has a predicted molecular mass of approximately 70 kDa (excluding the tag).

MBP-BAP31: The coding sequence corresponding to mouse B-cell receptor-associated protein 31 (BAP31, residues 63–245, UniProt Q61335) was cloned into the pMAL-C2X vector. The fusion protein contains an N-terminal MBP tag and has a predicted molecular mass of approximately 64 kDa (including the tag).

All constructs were verified by DNA sequencing. Proteins were expressed in E. coli and purified using affinity chromatography (glutathione Sepharose for GST-tagged proteins, Ni-NTA agarose for His-tagged proteins, and amylose resin for MBP-tagged proteins) according to the manufacturer's protocols. Purified proteins were dialyzed against storage buffer and stored at –80°C until use. Protein purity was assessed by SDS-PAGE and Coomassie blue staining, and identity was confirmed by Western blotting using anti-GST, anti-His, and anti-MBP antibodies, respectively.

Five groups were set up as follows. In groups 1–3, increasing amounts of MBP-BAP31 (150, 300, and 900 μg, respectively) were pre-incubated with a fixed amount (300 μg) of GST-STX17 on ice for 2 h. Subsequently, anti-GST magnetic beads and a fixed amount (300 μg) of His-ATG14 were added to each reaction, and the mixtures were incubated at 4°C with rotation for an additional 2 h. In group 4, anti-GST magnetic beads were incubated with GST-STX17 (300 μg) and His-ATG14 (300 μg) in the absence of MBP-BAP31. In group 5, anti-GST magnetic beads were incubated with GST-STX17 (300 μg) alone. Subsequently, the mixture was loaded onto Glutathione Sepharose 4B resin columns. After washing five times with wash buffer, proteins were eluted with wash buffer supplemented with 15 mM reduced glutathione. The eluates were separated using 12% SDS-PAGE, transferred to PVDF membranes (Millipore, Billerica, MA, USA), and probed with anti-GST, anti-His, and anti-MBP antibodies (Sigma-Aldrich, Merck KGaA, Darmstadt, Germany). GST, His6, and MBP proteins from Wuhan Genecreate (Wuhan, China) were used as negative controls. There were three replications for each pull-down assay.
