# Supplementary material for: BAP31 Drives Cartilage Calcification through Disruption of Autophagosome–Lysosome Fusion in Osteoarthritis
Source: Adv Sci (Weinh). 2026 Aug 29:e20140. Online ahead of print. doi: 10.1002/advs.202520140 (PMC13525426; doi:10.1002/advs.202520140)
Supplement: Supplementary file 2 [file ADVS-9999-e20140-s005.zip › Revised_Immunoblot uncropped images_advs.202520140.docx]

**Immunoblot uncropped images**

**Immunoblot uncropped images** Uncropped immunoblot images with molecular weights (in kilodaltons) labeled on left. CD63, Cluster of Differentiation 63; TUBULIN; ATG14, Autophagy Related 14; BAP31, B-cell receptor-associated protein 31; STX17, Syntaxin 17; CD81, Cluster of Differentiation 81; CD9, Cluster of Differentiation 9; LC3, Microtubule-associated protein 1 light chain 3; LAMP1, Lysosomal Associated Membrane Protein 1; and Calnexin; GST-STX17; HIS-ATG14; MBP-BAP31.
